# Mathematical models of developmental vascular remodelling: A review

**DOI:** 10.1371/journal.pcbi.1011130

**Published:** 2023-08-03

**Authors:** Jessica R. Crawshaw, Jennifer A. Flegg, Miguel O. Bernabeu, James M. Osborne

**Affiliations:** 1 Wolfson Centre for Mathematical Biology, Mathematical Institute, University of Oxford, Oxford, United Kingdom; 2 School of Mathematics and Statistics, The University of Melbourne, Melbourne, Australia; 3 Centre for Medical Informatics, The Usher Institute, University of Edinburgh, Edinburgh, United Kingdom; 4 The Bayes Centre, The University of Edinburgh, Edinburgh, United Kingdom; Stanford University, UNITED STATES

## Abstract

Over the past 40 years, there has been a strong focus on the development of mathematical models of angiogenesis, while developmental remodelling has received little such attention from the mathematical community. Sprouting angiogenesis can be seen as a very crude way of laying out a primitive vessel network (the raw material), while remodelling (understood as pruning of redundant vessels, diameter control, and the establishment of vessel identity and hierarchy) is the key to turning that primitive network into a functional network. This multiscale problem is of prime importance in the development of a functional vasculature. In addition, defective remodelling (either during developmental remodelling or due to a reactivation of the remodelling programme caused by an injury) is associated with a significant number of diseases. In this review, we discuss existing mathematical models of developmental remodelling and explore the important contributions that these models have made to the field of vascular development. These mathematical models are effectively used to investigate and predict vascular development and are able to reproduce experimentally observable results. Moreover, these models provide a useful means of hypothesis generation and can explain the underlying mechanisms driving the observed structural and functional network development. However, developmental vascular remodelling is still a relatively new area in mathematical biology, and many biological questions remain unanswered. In this review, we present the existing modelling paradigms and define the key challenges for the field.

## Author summary

In this review article, we examine the existing models of developmental vascular remodelling and discuss the many contributions these models have made to the field of vascular development. Four individual modelling paradigms are identified, and future directions for each paradigm are addressed. The four modelling paradigms we identify are as follows: (1) logarithmic models of vascular calibre determination; (2) hybrid models of vascular regression and calibre determination; (3) cell-based models of vascular remodelling; and (4) flow-focused models of regression. Finally we identify the development of multiscale models, subcellular models, and experimental model validation to be the major challenges for the field moving forward.

## 1 Introduction

**Fig 1:**
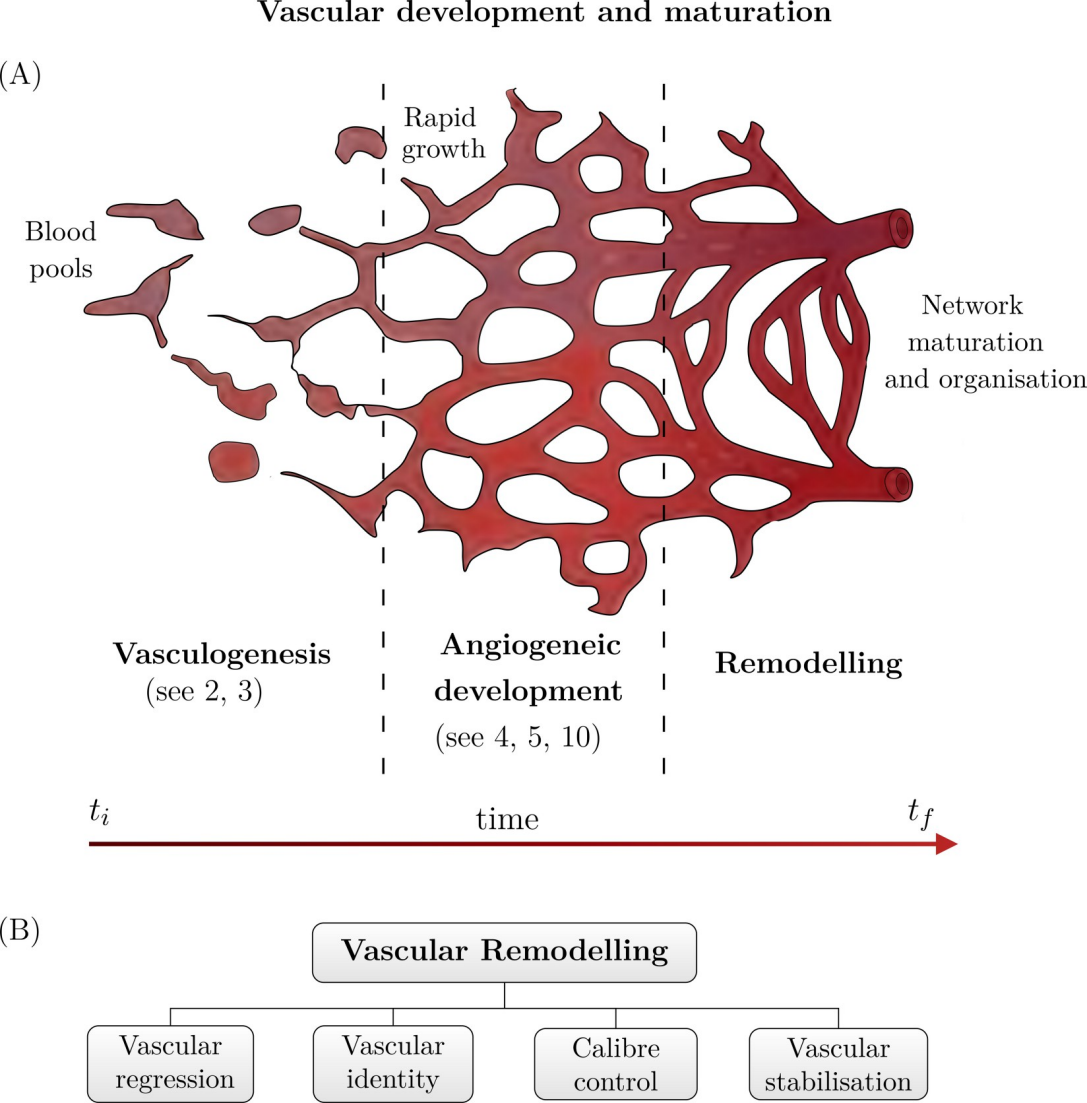
Schematic of vascular development. (**A**) Each stage of development is shown from left to right. On the left, the formation of blood islands join together to form the initial components of the vascular network (vasculogenesis). This initial network is rapidly extended upon during angiogenesis, creating a dense unstructured network (middle). Finally, this dense network organises and matures during remodelling and regression (right). (**B**) Vascular remodelling can be subdivided into several distinct categories: vascular regression, vascular identity formation, calibre control, and vascular stabilisation. The papers cited in this figure are [2–5,10].

Vascular remodelling describes a critical set of developmental processes that drive vascular network maturation and concludes vascular development [1]. The primitive vascular networks established through vasculogenesis [2,3] and angiogenesis [4,5] are immature, unorganised, and have widespread redundancy (Fig 1A) [1,6]. Following (and in coordination with) angiogenic development, the primitive vasculature is reorganised and matures through vascular remodelling [6], which encompasses vascular regression, vascular identity determination, dynamic calibre (diameter) control, and vascular stabilisation (Fig 1B) [1,6]. Vascular remodelling occurs throughout life in both physiological and pathological contexts [1]; however, in this review, we will be focusing on developmental remodelling. Note, calibre control in the context of homeostasis and disease has been extensively studied, however, is not considered in this development-focused review.

Alongside experimental techniques, the use of mathematical models has been integral in the advancement of our understanding of developmental vascular remodelling. There is, however, of course, still much we do not know. Many critical pathways and processes are only now coming to light with recent advancement in both experimental technology and computational power. The fundamental role of mathematical biology in the field of developmental vascular remodelling is continuing to gain increasing recognition and will play a pivotal role in advancing the field moving forward.

**Fig 2:**
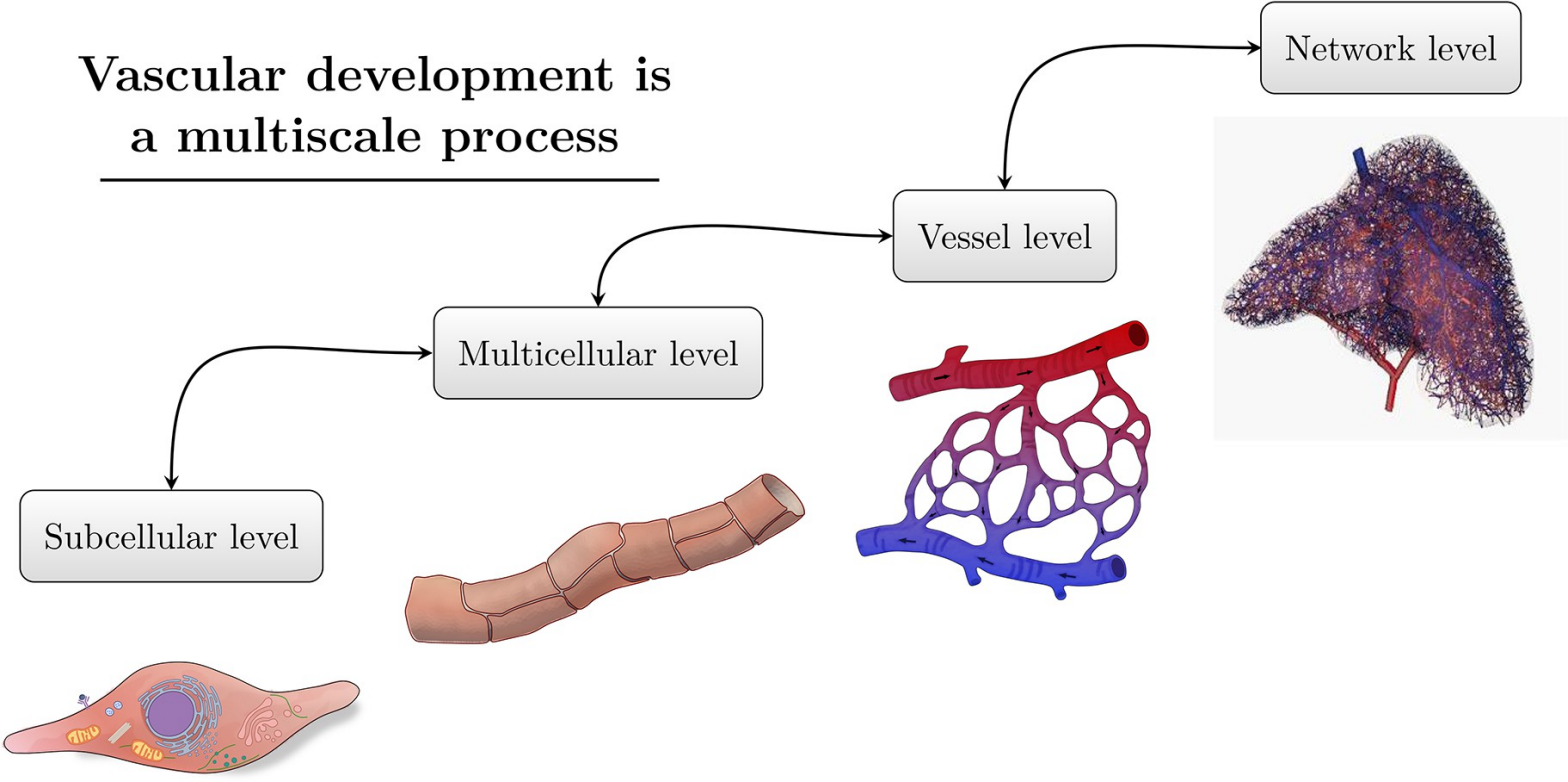
Vascular remodelling is a multiscale process. Vascular remodelling should be considered from a multiscale approach, considering the subcellular (left), multicellular (second across), vessel level (third across), and network left (right). The 3D printed vascular network shown on the far right has been reproduced with permission from [11].

Vascular remodelling is a multiscale process (Fig 2). The activities of the endothelial cells drive the essential reorganisation and restructuring of the network to produce a functional vasculature. During each component of remodelling, endothelial activity is driven by mechanical stimulus from the local blood flow, cellular signalling, and genetic factors, occurring at a subcellular level [1,7–9]. As such, the development of detailed models of vascular remodelling requires a multiscale and multiphysics approach. In the following subsections, we provide a brief overview of the key components of vascular remodelling, before detailing the mathematical literature in Sections 2 to 6.

### 1.1 Vascular regression

Vascular regression is a key developmental process occurring during remodelling in which select capillaries are removed from the network [12]. Regression can be subdivided into 2 distinct categories: (**1**) **pruning**: the removal of selected capillaries in order to create an optimised vascular network; and (**2**) **involution**: the removal of an entire capillary bed [1]. The cellular and mechanical mechanisms driving regression vary significantly between different vascular networks. Moreover, pruning and involution are achieved through distinct developmental pathways, as discussed in the following paragraphs.

#### Vascular pruning

**Fig 3:**
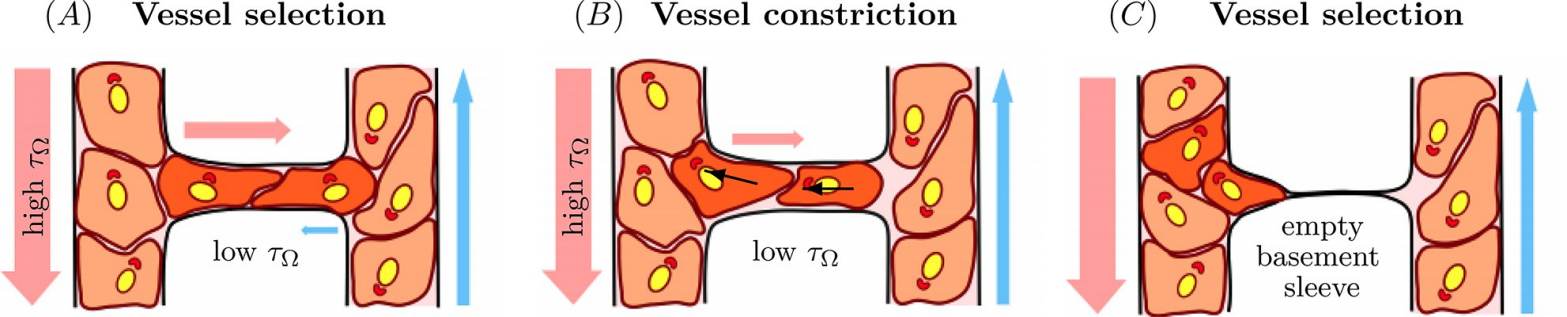
Schematic of vascular pruning. (**A**) Initial selection of a vessel with low wall shear stress, ${\vec{\tau }}_{\Omega }$. (**B**) The endothelial cells from the pruning branch polarise and migrate up the wall shear stress gradient and against flow towards the neighbouring vessel, as shown by the black arrows. (**C**) The endothelial cells of the pruning branch are incorporated into the neighbouring vessel, leaving an empty basement sleeve in place of the pruning vessel. The coloured arrows indicate the direction and the intensity of blood flow, with arterial flow shown in red, and venous flow shown in blue. The endothelial cell nuclei and the Golgi apparatus in within each cell are yellow and red, respectively, showing the endothelial cell polarisation against the flow. Figure adapted with permissions from Santamaría and colleagues [12].

Recent studies have shown that pruning is primarily driven by active endothelial cell migration triggered by the wall shear stress imposed on the vessel by the local haemodynamic environment [13,14] (Fig 3). In these studies, it was shown that endothelial cells actively migrate away from vessels with low wall shear stress and low-flow, towards neighbouring vessels with high-wall shear stress and high-flow [7,13]. This directed migration of endothelial cells from the low-flow vessels towards the high-flow vessels causes a destabilisation and collapse of the former and a stabilisation of the latter, thereby resulting in vascular pruning and network remodelling [12–17]. Much of our understanding around the role of wall shear stress during pruning has been gained through hybrid experimental and computational fluid dynamics studies [7–9,13,14,18] and is discussed in Section 5 in detail. Current research suggest that pruning occurs in 3 (or 4) distinct steps: (1) vessel selection; (2) vessel collapse; and (3) resolution, as shown in Fig 3 [6,12]. The endothelial subcellullar machinery underlying blood flow sensing (mechanosensing) is complex and is still being deciphered [12]. It remains unclear how mechanosensing translates into cellular responses such as polarisation, alignment, and direction migration to drive pruning and other remodelling processes.

In conjunction with the haemodynamic environment, vascular pruning is driven by a critical balance of cellular signalling pathways, particularly, vascular endothelial growth factor (VEGF), Wnt signalling, Delta/notch signalling, and angiopoietin [6,8,19–25]. Similar to the vascular response to the local haemodynamic environment, the roles of each cellular pathway are still being deciphered, although we know they are largely context dependent, and vary between networks, developmental stages, and between pathologies [6]. The overexpression of VEGF promotes endothelial cell survival signalling, thereby impeding retinal vascular regression [26,27]. Conversely, down-regulation of VEGF results in excessive endothelial cell apoptosis and vessel regression in the developing retinal network [28,29]. Angiopoietin has critical roles in vessel formation and remodelling. Angiopoietin-1 signalling promotes endothelial cell survival and quiescence [21], whereas autocrine angiopoietin-2 signalling promotes vessel destabilisation and regression [22,30]. Interestingly, in VEGF-rich environments, angiopoietin-2 promotes endothelial cell migration, sprouting, and proliferation [22,31]. In the retinal and embryonic networks, Wnt signalling acts to modulate vessel regression, while disruption of the Wnt signalling pathways manifests in premature and excessive regression [23]. However, in other networks, such as the hyaloid vasculature, Wnt, in conjunction with angiopoietin-2, promotes vessel regression [32]. The endothelial cell sensitivity to shear stress has been shown to be moderated by noncanonical Wnt signalling [8]. Moreover, cross talk between the Wnt/*β*-catenin pathway with the Delta/Notch pathway is critical for postnatal retinal vessel stabilisation [33]. Conversely, during vasoconstriction and vessel occlusion, Delta-like 4/Notch signalling promotes vessel regression [20,34].

Endothelial cell apoptosis (death) is though to have a minimal role during vascular pruning. It is thought endothelial cell apoptosis is triggered by the loss of survival signalling factors and local macrophage activity [14,15]. Finally, pruning can be also achieved through reverse intussusception, during which the endothelial membrane from opposing sides of the vessel lumen fuse together, thereby occluding the vessel and ensuring a controlled closure of a regressing vessel free of blood leakage [35].

Vascular pruning is a multiscale developmental process driven by endothelial cell activity. While there is an absence of both subcellular and multiscale models of vascular pruning in the literature, there are several models considering vascular pruning at a network level (hybrid models, Section 3) and at the cellular level (flow-focused models, Section 4). These models often conflate pruning with calibre determination, making the false assumption that pruning is a by-product or endpoint of calibre determination. For a detailed review of vascular pruning, see Santamaría and colleagues [12] and Korn and colleagues [6].

#### Vascular involution

Vascular involution refers to the ablation of the transient capillary beds, such as the hyaloid network and the pupillary membrane [1]. Involution is controlled through developmental programs that orchestrate a domino-like elimination of the network in 2 phases: (1) generalised vascular stenosis, which incites (2) a synchronised wave of endothelial cell apoptosis due to the flow disturbances [10,36–38]. Apoptosis is thought to trigger the initial vascular stenosis in the hyaloid vessels [39]; however, the trigger for the initial stenosis in other networks is debated [1,40,41]. Involution also occurs post-development (throughout life) in transient capillary beds, such those supplying the ovarian follicles and mammary glands, due to the decreasing metabolic demands of the tissue [42,43]. While vascular involution is an important stage of ocular development, involution has yet to be considered from a mathematical lens.

### 1.2 Vascular identity determination

The specific vessel type and organotypic identity is determined by the phenotype of the endothelial cells composing the vessel wall, which is established during remodelling [1,44]. As vessel structure and function varies greatly across the vascular system, specialised remodelling processes are required to achieve vascular maturity, stability, and functionality for each vessel type [45]. These specialised remodelling processes are controlled by endothelial cells phenotype, which dictates the cellular response to external stimuli (such as wall shear stress and VEGF) and the specialised subcellular processes necessary to forge these specialised remodelling pathways. As such, it is necessary to understand endothelial phenotype and phenotype determination to build a multiscale model of developmental vascular remodelling. Recent advance in experimental techniques, such as single-cell RNA sequencing, have rapidly extended our understanding of the mechanisms controlling phenotype determination and network organisation [1,46]. Although there is still much left to understand, from recent studies, it is clear that a range of genetic factors and cellular signalling factors are the primary mechanisms regulating phenotype determination, while blood flow provides an auxiliary role [47–51]. The genetic and signalling factors dictating arterial and venous specification are vastly different, and far more is understood about arterial specification.

Currently, there is a single model in the mathematical literature considering vascular identity determination [52]. This sole study investigates the interaction between endothelial cells and recruited smooth muscle cells during arterial specification. At present, there is an absence of models examining the factors controlling phenotypic determination. The development of new mathematical models to understand how the interdependent genetic pathways and signalling factors interact to dictate endothelial cell phenotype, and thereby vascular identity, will be an important step forward in understanding the biological processes controlling remodelling.

### 1.3 Calibre determination

The diameter of each vessel is established during development and adapts throughout life to meet both physiological and pathological needs. The establishment of an optimal calibre (diameter) throughout the vasculature helps produce a hierarchical network that provides energy-efficient blood transport and ensures that perfusion matches the local tissues irrigation requirements [1]. While the mechanisms through which vessels attain an optimised diameter are still largely unknown, blood flow has been recognised to play a key role [16,53]. Moreover, there are studies emerging to suggest that the role of the blood flow is regionally attenuated and amplified by the local cellular signalling factors. [54,55] As such, the processes driving calibre determination vary between vessel types and between networks [1]. Evidence provided by a hybrid experimental-mathematical study suggests that the determination of arterial diameter is driven by endothelial cell proliferation and directed migration (from low to high wall shear stress) and vessel fusion [56]. Conversely, it is thought that endothelial cell apoptosis regulates capillary diameter during maturation [57]. Calibre control to maintain homeostasis and during disease has been extensively studied, however, is not considered in this review; for details, see Ramanlal and Gupta [58] and Kellogg and colleagues [59].

The first mathematical models of remodelling, published by Pries and Secomb [60–62], concentrated on vascular calibre determination, however, blurred the lines between developmental, homeostatic, and pathological regulation of vessel diameter. These model assumed that a logarithmic relationship exists between the vessel diameter and the wall shear stress, pressure, and metabolic/signalling factors. Using these assumptions, it was shown that that a hierarchical network could evolve from a uniform network [60–62]. Unfortunately, there is an absence of models in the literature considering the cellular, genetic, and mechanical processes driving vessel calibre determination. Moreover, there are no models examining the synergistic relationship between blood flow and signalling factors during calibre determination. Development of such a model will prove important in understanding the heterogeneous determination of vessel diameter across the vascular system (between different vessel types and different vessel beds). Moreover, such a model will provide an accurate model of hierarchical optimisation and optimised blood flow. Note, calibre control in the context of homeostasis and disease has been extensively studied, however, is not considered in this development-focused review.

### 1.4 Vascular stabilisation

During the final stages of vascular development, the newly established vessels must stabilise while the endothelial cells attain a homeostatic metabolic state [1,63]. The manner in which vascular stabilisation is achieved is dependent on location and vessel type. For capillaries, broadly speaking, stabilisation is achieved through the recruitment of pericytes and formation of the basement membrane [64]. Vascular stabilisation has been partially addressed by Peirce and colleagues [52] however has yet to be fully explored in the mathematical literature. The roles of basement formation and endothelial cell metabolism during remodelling has yet been considered by the mathematical community.

### 1.5 The importance of mathematical models of vascular remodelling

As discussed above, the development and analysis of mathematical models of vascular remodelling has been essential in the advancement of our understanding of vascular development. These models have been successfully used to examine, understand, and anticipate vascular development. Moreover, these models provide a useful means of hypothesis generation and can explain the underlying mechanisms driving the observed structural and functional network development. There is, however, much we do not understand about remodelling, and the development of mathematical models will help fill these gaps.

In the following sections, we will consider mathematical models of developmental vascular remodelling. The models of remodelling currently available in today’s literature can be broadly categorised into 4 paradigms;

**Paradigm 1**: Logarithmic models of calibre determination
**Paradigm 2**: Hybrid models of regression and calibre determination
**Paradigm 3**: Multicellular models of remodelling
**Paradigm 4**: flow-focused models of regression.

**Fig 4:**
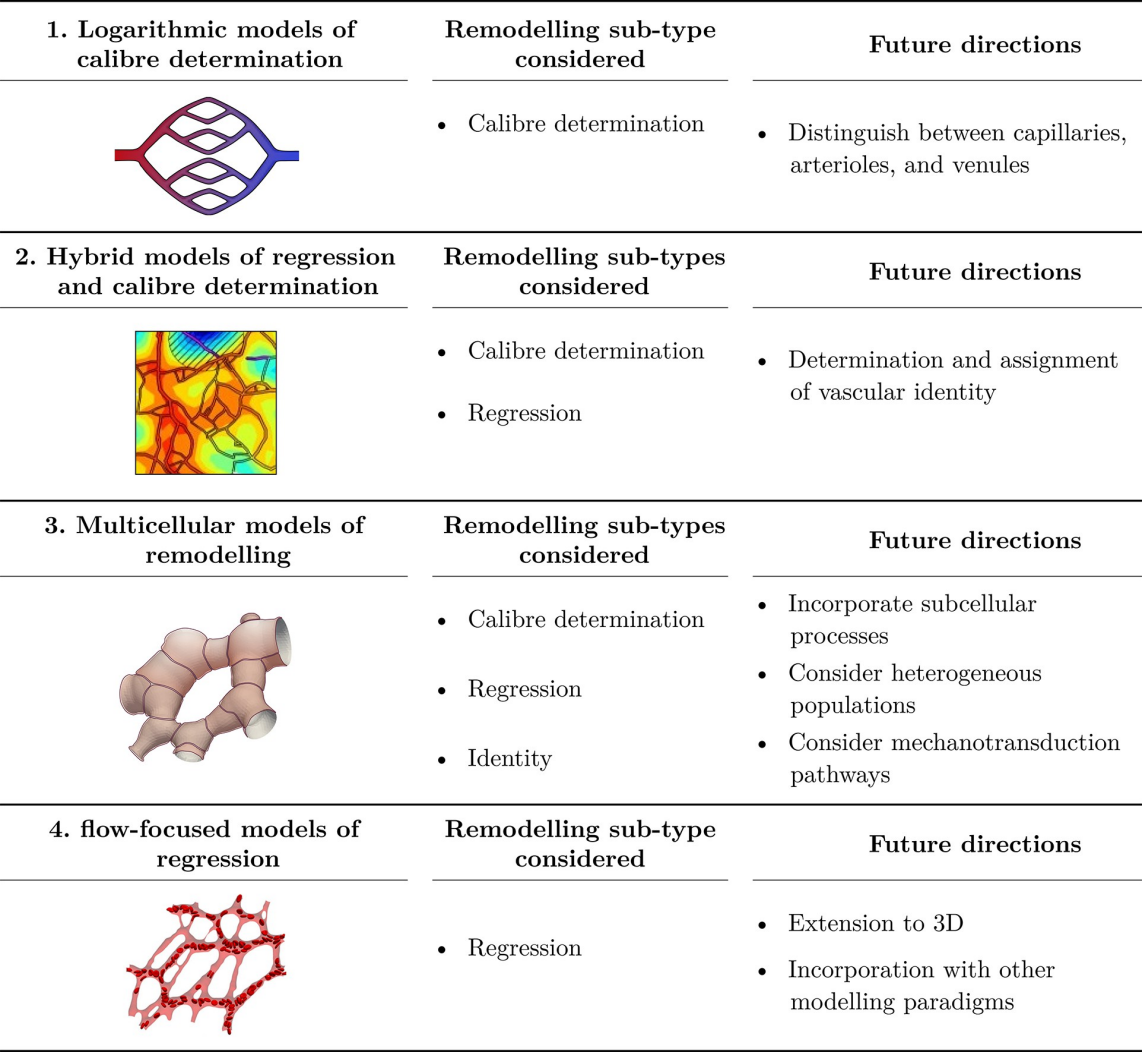
Summary of future directions for each modelling paradigm and the remodelling subtypes addressed by each paradigm. The simulation shown in the left column of row 2 has been reproduced with permission from Secomb and colleagues [65]. The network shown in the left column of row 4 has been reproduced with permission from Zhou and colleagues [66].

**Fig 5:**
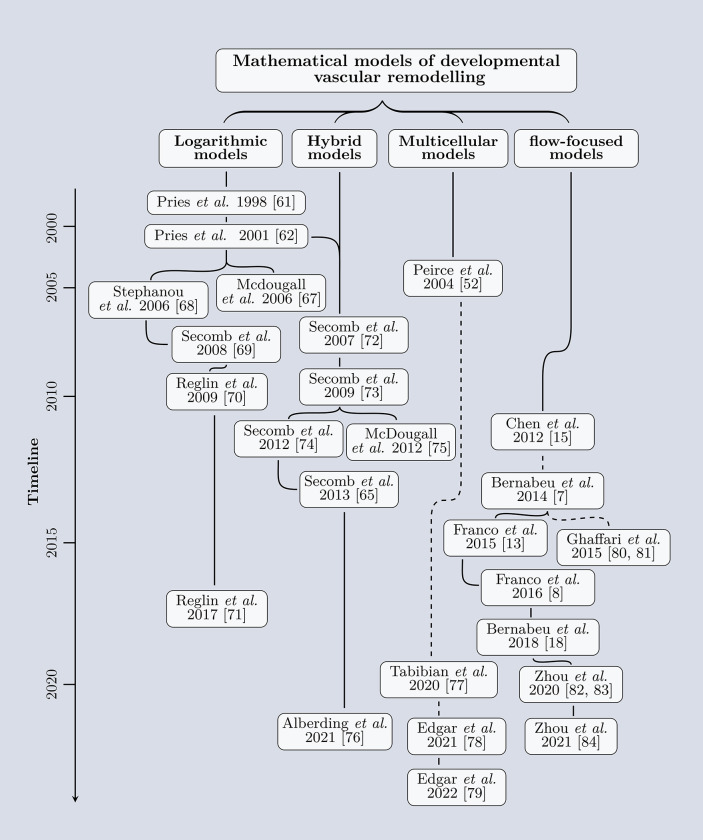
A summary of the currently available models of vascular regression and remodelling. Models of vascular regression and remodelling can be broadly categorised into 4 paradigm: logarithmic models, hybrid model, multicellular models, and flow-focused models. Related models are connected by a solid black line, while unrelated models that are subsequent on the timeline are connected with a dashed black line. A (nonlinear) timeline is shown on the left. The papers cited in this figure are [7,8,13,15,18,52,61,62,65–83].

A visual depiction of each paradigm is shown in Fig 4 (leftmost column). Each paradigm has been developed to specifically consider a single, or several, remodelling subtypes, as shown in the middle column of Fig 4. The publications associated with each paradigm can be seen in Fig 5 and are discussed in detail in the following sections below, along with the important future directions for each paradigm (Fig 4: rightmost column).

## 2 Modelling paradigm 1: Logarithmic models of vascular calibre determination

**Fig 6:**
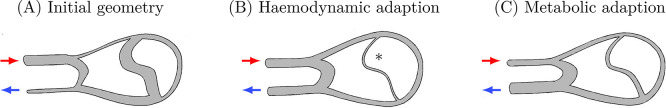
A sample logarithmic model of vascular calibre determination in which the vessel diameter is logarithmically dependent on the local haemodynamic factors (B) or the metabolic stimuli (C). The initial network configuration is shown in (**A**) in which the vessels initially have a random diameter with no adaption. (**B**) When the vessel diameter is controlled by the local wall shear stress, the diameter of the anastomosis (vessel denoted by the asterisk) decreases in response to the comparatively lower wall shear stress. (**C**) The diameter of each vessel is responsive to metabolic stimuli, which acts to stabilise the diameter of each vessel in this model. Figure reproduced from Pries and colleagues [62] with permission. Copyright 2001 the American Physiological Society.

The earliest model of vascular remodelling was published by Pries and colleagues [61] in 1998 (Fig 5), who considered the additive roles of wall shear stress, haemodynamic pressure, and local metabolic factors on vascular calibre determination. Pries and colleagues [61] assumed that the vessel diameter was logarithmically dependent on each of the aforementioned factors, and using this basic assumption was able to predict and reproduce the experimentally observed hierarchical organisation seen in simple vascular networks (Fig 6). While logarithmic models were originally developed to consider adaptive remodelling, this model is sufficiently general that it is often applied to developmental remodelling and is considered by many to be the foundation model of vascular remodelling. This model is heavily cited and is core to many of the subsequent models of regression and calibre determination [65,67–76].

In the original remodelling paper by Pries and colleagues [61], the expression relating the vessel diameter (*D*) with wall shear stress (*τ*_*w*_), pressure (*P*), and local metabolic factors was given by

$$\Delta D=(\underset{\begin{array}{c} Wallshear\\ stressdependence \end{array}}{\underset{︸}{log(\tau_{w}+\tau_{ref})}}-\underset{\begin{array}{c} Pressure\\ dependence \end{array}}{\underset{︸}{log(\tau_{e}(P))}}+\underset{\begin{array}{c} Metabolic\\ dependence \end{array}}{\underset{︸}{k_{m}log(\frac{Q_{ref}}{QH_{D}}+1)}}-k_{s})\frac{D\Delta t}{t},$$ (1)

where *t* is time, *τ*_ref_ is the reference wall shear stress, *Q* and *Q*_ref_ are the local and the reference fluid flow, respectively, *H*_*D*_ is the hematocrite concentration, and *τ*_*e*_(*P*) is a function relating the pressure to an expected wall shear stress. Capillary “sensitivity” to the local metabolic state is given by *k*_*m*_, and the “shrinking tendency” of the vessel is defined by *k*_*s*_. The logarithmic dependence provided in Eq (1) was a modelling choice intended to prevent large changes in the vessel diameter for large values of wall shear stress and pressure [76]. In their original paper, the authors acknowledge that their model involved several assumptions that could not be justified experimentally at the time; however, the purpose of the model was to provide a starting point for this emerging branch of mathematical vascular biology. This purpose has been satisfied, with variations of this equation forming the mathematical foundation of the hybrid modelling paradigm reviewed in Section 3. In recent years, the predicted logarithmic relationship between vessel diameter and wall shear stress has been experimentally observed in the developing vasculature of zebrafish [15], though a similar relationship between pressure, metabolic signals, and vessel diameter has yet to be reported. Moreover, the assumption of a constant reference wall shear stress (*τ*_*ref*_) throughout the cardiovascular system was recently put into question by the set-point theory of the Schwarz lab [84–87]. The expression for the wall shear stress dependence shown in Eq (1) has been maintained in subsequent iterations and implementation of logarithmic models; however, both the pressure and metabolic dependence have been updated in the years following this original publication [62,76].

Based on experimental data obtained in the rat mesentery, Pries and colleagues [61] initially suggested that the relationship between expected wall shear stress and pressure (given in Eq (1)) should take the sigmoidal form of

$$\tau_{e}(P)=100-86exp(-5,000[log(log(P)){]}^{5.4}).$$

This approach by Pries and colleagues [61] has proven very popular over the years, however, was recently updated by Alberding and colleagues [76] who utilised a fitted Hill function to describe the relationship between pressure and the expected wall shear stress:

$$\tau_{e}(P)=14+86(\frac{P^{5}}{P^{5}+36^{5}}).$$

Alberding and colleagues [76] went further to modify Eq (1), introducing an assumption that the local metabolic dependence and the conducted metabolic signal were also inversely dependent on the vessel diameter. This adaptation was introduced to prevent excessive regression of smaller vessels from the network, while also preventing unrealistically large arterioles and venules.

Several years following the publication of Pries and colleagues [61], the authors published an updated expression for the dynamic vessel diameter, in which the metabolic component of Eq (1) was adapted to account for both the local and conducted metabolic signals [62]. The expression for the local metabolic signal follows a simple logarithmic dependency with the local oxygen concentration. It was assumed that the local oxygen availability would characterise metabolic conditions in the local tissue, and, as such, the consideration of oxygen alone would produce a sufficient representation of the local metabolic signal. The conducted metabolic signal, described using a Hill function, was designed to mimic the communication between the endothelial cells lining the vessel [62] and is one of the few expressions in this field of mathematical biology to explicitly consider this pathway during vascular remodelling. This adaptation to the original expression proposed by Pries and colleagues [61] provided a more realistic expression for vascular calibre determination and allowed the authors to simulate more biologically realistic networks.

Logarithmic models of vascular remodelling were the first mathematical models to consider vascular remodelling, and, as such, simplifying assumptions were necessary. In each variation of the logarithmic models discussed above, structural and functional distinctions between capillaries, arterioles, and venules are blurred in the interest of simplicity. These models assume all vessels respond in an arteriole-like manner. As discussed in Section 1.3, the mechanisms, though which each vessel type attains the necessary diameter, are complicated and distinct. It is thought that endothelial cell migration dictates calibre determination during development. As such, the relationship between stimuli and capillary diameter is likely to be discontinuous and highly nonlinear. Further developments on this modelling paradigm should consider vessel identity, distinguishing between arterioles, capillaries, and venules to establish a more biologically accurate model of capillary response (direct or indirect) to each stimulus (Fig 4: Row 1).

Logarithmic models of vascular remodelling have provided an important proof of concept, showing that if we assume wall shear stress, pressure, and metabolic stimuli control vessel diameter, we see the formation of a primitive hierarchical structure. Experimental data are now emerging to suggest that the change in vessel diameter during remodelling is proportional to the natural logarithm of the wall shear stress [80]. Moreover, the relationship between vascular regression and wall shear stress has since been established by experimental and flow-based studies [8,12,13,77]. As such, it is now necessary to establish a data-driven relationship between the changing vessel diameter, pressure, and metabolic stimuli. Since the introduction of this relationship between capillary diameter and local stimuli, Eq (1) has enjoyed widespread use and has been co-opted in the development of hybrid models of vascular development and regression [65,72–76], as discussed in the following section.

## 3 Modelling paradigm 2: Hybrid models of vascular regression and calibre determination

Over the past 14 years, the Secomb group have developed a hybrid model of vascular development, in which a biologically realistic capillary plexus emerges from an initial collection of vessel segments [65,72–74,76] (Fig 5). Hybrid models integrate a discrete and continuum modelling approach and inherit principles directly from logarithmic models to explicitly consider the importance of remodelling alongside angiogeneic development in evolving vascular networks.

In these models, the capillary network is usually defined by an evolving network of discrete 1D rigid line segments that extend into an avascular tissue according to the

1. Blood flow at each line-segment;
2. Local concentration of growth factors; and
3. Local oxygen concentration.

The local concentration of oxygen and growth factor are modelled as continuums and are calculated using advection-diffusion equations, while the flow is assumed to be Hagen–Poiseuille flow and calculated using a network-based model at each time step. Based on the above factors, the network of 1D line segments expand according to the following 4 rules:

**Rule 1**: Sprouts form in regions of high growth factor concentration in a probabilistic manner and grow at a constant rate;
**Rule 2**: Tension in the vessel and the underlying tissue dictate the direction of sprout elongation;
**Rule 3**: Vessel diameter varies according to the logarithmic model in Eq (1); and
**Rule 4**: If a vessel falls below 3 *μm* in diameter, it is pruned from the network.

By coupling the classic rules for angiogeneic development (Rules 1 and 2) with the equation for vascular calibre determination from Pries and colleagues [62] (Rule 3), and the new regression rule (Rule 4), the Secomb group created a model that captures the rapid vascularisation of the avascular tissue, followed by remodelling of the network and regression of the redundant vessels. Indeed, it was shown [65] that the steady state of this evolving network in 2D replicates a hierarchical network, with vascular density, vessel length, and bifurcation angles in good agreement with experimental results [88].

**Fig 7:**
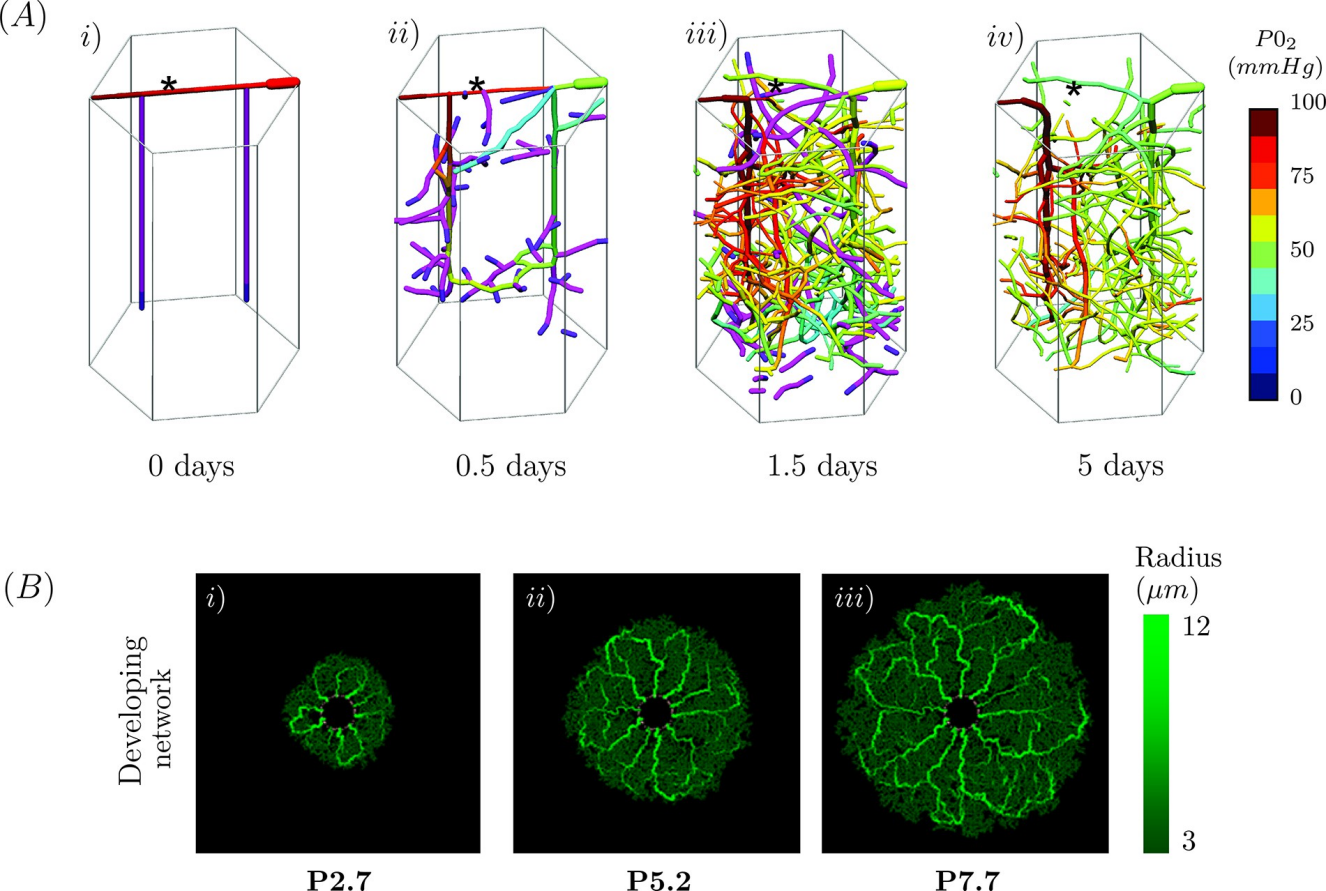
Hybrid models of vascular development. (**A**) Alberding and colleagues [76] presented the first 3D hybrid model of vascular development in the cerebral cortex. The initial simulation configuration is shown (i) from which vessels angiogenically sprout (ii) to from a dense network (iii) with variation in vessel diameter. Finally, vessels falling below a given diameter threshold are removed from the network to form an optimised cortical vasculature (iv). In each figure, the partial pressure of oxygen is coloured according to scale at the right in (iv). (**B**) McDougall and colleagues [75] adapted the Secomb hybrid model to simulate retinal vascular development, presentation development at P2.7 (i), P5.2 (ii), and P7.7 (iii). The green shading of each vessel depicts the vessel radius, with larger vessels shown in lighter green, and smaller vessels shown in darker green (scale on right). Subfigure A reproduced with permission from Alberding and colleagues [76], and subfigure B reproduced with permission from McDougall and colleagues [75].

In a more recent publication, Alberding and colleagues [76] extended this model into 3D using a periodic 3D hexagonal prism to study the development of the cerebral cortex vasculature (Fig 7A). Again, this extended model demonstrated an initial growth phase (Fig 7Ai-ii), followed by a period of remodelling to produce an optimised network (Fig 7Aiii-iv). The structure and density of the networks produced by the Alberding and colleagues [76] model are very similar to the cortical vasculature observed in mice [89,90] and humans [91,92]. Moreover, this model replicates the transition from a planar (2D) network to a 3D network that is seen experimentally in developing cortical vasculature networks [93]. Following the establishment of a biologically realistic baseline, Alberding and colleagues [76] used this model to study how pathological perturbations to physiological development generated dysfunctional development and disease. The results from this model added weight to previously hypothesised disease pathways [61,65].

McDougall and colleagues [75] introduced several adaptations to the Secomb model [65,72–74,76] to examine retinal vascular development. In their model, the vascular network is expressed as a series of nodes over a 2D square lattice (as opposed to a series of connected 1D line segments), which evolve iteratively via 2 continuous partial differential equations describing the density of astrocytes and endothelial cells at each nodal point. To date, there is only one other model to also consider the supporting role of the surrounding cell populations during vascular remodelling [52]. While the Secomb model allows for the network to extend in any direction, the McDougall model restricts movement to the axial directions and introduces the freedom random moment. In a departure from the Secomb modelling framework [65], the McDougall and colleagues [75] model considered the endothelial cell density at each node. This provided an opportunity for the authors to adapt Eq (1) to consider the role of endothelial cell density on the vessel radius and propensity to regress; however, this was not done. This adaption may produce a more biologically faithful description of vascular development and provides an interesting avenue for future work.

A sample of a simulated retinal vasculature is shown in Fig 7B, where we see vessels developing radially outwards from the optic nerve (Fig 7Bi) and forming a dense circular vasculature (Fig 7Bii-iii). This model successfully recreated the regression experimentally observed around the arterioles at the optic nerve; however, the extent of regression is not reflective of that reported in experimental studies by postnatal day 7 [94]. As such, although the arteriole radii are notably larger than that of the surrounding capillaries, the formation of a hierarchical network is not achieved by postnatal day 7.7 [94].

A notable strength of the McDougall model is its incorporation of 2 different cell populations and its inclusion of multiple cellular signalling factors. This is, again, another departure from the Secomb framework where a single generic “growth” signalling factor is considered. This extension of the basic hybrid model demonstrates the ease with which additional and more specialised signalling factors (be it chemical, cellular, or mechanical) can be integrated into the model. Indeed, this is a major strength of the hybrid continuum-discrete model, providing a relatively simple means of modelling vascular development across several scales (multicellular, vessel, and network scales). It is, however, important to keep in mind that these models assume all vessels respond in a homogeneous manner, with the cellular and subcellular processes diluted to a simple uniform response. The inclusion of a more comprehensive system of signalling factors will provide a conceptually simple and interesting future extension of this model. However, practical challenges in implementation and parameterisation will likely arise due to the interconnected and dependent nature of many signalling factors and stimuli. Such an extension would be useful to improve our understanding of how noncanonical Wnt and angiopoietin-2 signalling can control vascular regression [22,30].

It should be noted that since the hybrid modelling paradigm inherits its foundational framework from logarithmic models, it has also inherited the key modelling weakness, namely the lack of distinction between vessel types. Again, this weakness could be remedied by providing biologically accurate vessel type–specific response to stimuli. Moreover, hybrid models are uniquely posed to study vessel identity determination as they consider both chemical and mechanical driven remodelling at the level of each vessel while considering development at the network scale. As such, consideration of vessel identity will be crucial in improving our understanding of the biological processes determining vascular identity (Fig 4: Row 2). Experimental studies are only just beginning to shed light on the complex processes governing vascular identity determination, and mathematical models will play a key role in deciphering this developmental process [1].

Hybrid models have been adapted and simplified in the modelling of tumour vascularisation. These models adopt the model rules described above and weight the incidence of remodelling events probabilistic manner. These stochastic adaptions of the hybrid model are embedded within larger stochastic models of tumour development, and, as such, computer efficiency and model simplicity is a priority.

While logarithmic and hybrid models have provided a set of rules capable of imitating processes occurring on the cellular and vessel scales, these modelling paradigms do not consider the cellular behaviour that drives vascular regression. To do so requires the implementation of a multicellular model of regression, such as those discussed in the next section.

## 4 Modelling paradigm 3: Cell-based models of vascular remodelling

**Fig 8:**
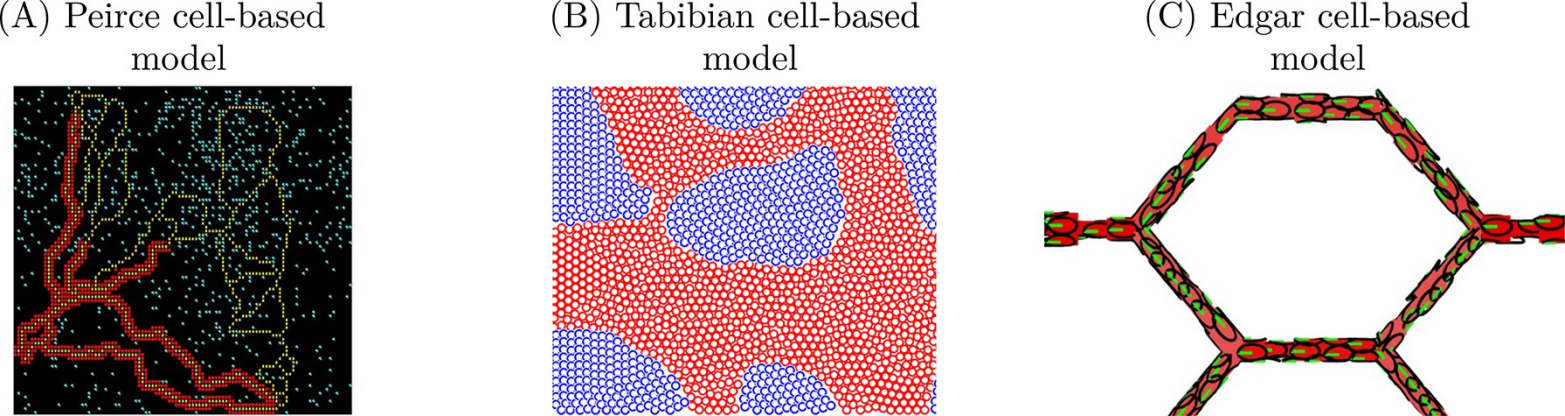
The Peirce (A), Tabibian (B), and Edgar (C) models are the 3 cell-based models currently available in the literature. (**A**) The Peirce and colleagues [52] cellular automaton model examining vascular stabilisation and identity determination; those vessels composed of endothelial cells (red) surrounded by smooth muscle cells (yellow) are becoming arterioles, while the vessels composed of only endothelial cells (yellow) are still capillaries. (B) Tabibian and colleagues [77] published a self-propelled particle model of vascular calibre determination; red circles are components of the capillary, and blue circles are components of the surrounding tissue. (C) Edgar and colleagues [78,79] developed a sphere-based model of vascular calibre determination and regression; each endothelial cell is depicted by an ellipse with the polarity given by the green line. Subfigures A, B, and C are reproduced from Peirce and colleagues [52], Tabibian and colleagues [77], and Edgar and colleagues [79], respectively, with permissions.

Only very recently have cell-based modelling techniques been used to examine the cellular behaviour driving vascular remodelling (Fig 5). Cell-based models are a useful tool with which to unravel the complex nonlinear interactions between cells and between processes at the subcellular and multicellular scales [95]. Such discrete approaches are very useful when modelling the inherently discrete cellular and subcellular processes driving vascular development and remodelling. Moreover, cell-based approaches introduce an attractive opportunity to consider heterogeneities across the endothelial cell population (which controls the response to stimuli), to consider a larger cohort of the different cellular populations involved in coordinating vascular remodelling, and to investigate the cellular building blocks of certain emerging phenomena. Cell-based models (also referred to as discrete-based, or agent-based models) have been successfully used in many other areas of mathematical biology [95] and have been particularly popular in the study of angiogenesis [96]. Currently, the Peirce model [52], the Tabibian model [77], and the Edgar model [78,79] are the only cell-based models of vascular remodelling available in the literature (Fig 5). Each of these models adopts a different cell-based approach and considers a different aspect of remodelling. These models are shown in Fig 8 and discussed in the following paragraphs.

The first to consider vascular remodelling using a cell-based model was Peirce and colleagues [52], who used a cellular automaton model to simulate the angiogenic growth and arterial specification of an expanding vascular network. In this model, the authors explicitly considered endothelial cells and smooth muscle cells (Fig 8A). Endothelial cells were considered to secrete platelet-derived growth factor-BB (PDGF-BB), which acted to stimulate smooth muscle cell proliferation and migration. When the blood flow–induced wall strain exceeded a predefined threshold, the rate of PDGF-BB expression increased. Following these simple rules, smooth muscle cells were shown to extend downstream along the vessel and were recruited from the surrounding tissue as the existing network angiogenically expanded. As such, the newly formed vessels in this model attained a functional identity of either an arteriole or venule, surrounded by smooth muscle cells, or capillary, devoid of smooth muscle cells (Fig 8A). Arterial specification was quantified in terms of the increase in length density of smooth muscle covering vessels segments and was shown to be very similar to in vivo data. Although the importance of this model was not fully appreciated at the time, this is the first model of vascular remodelling to consider vascular identity. Moreover, this is the first model of remodelling to consider multiple cellular populations and remains the only to consider the importance of smooth muscle cells. Further work is needed in this area to develop a more nuanced approach in which endothelial cell phenotype dictates smooth muscle cell recruitment, and the genetic factors determining endothelial cell phenotype and vascular identity are considered.

Two decades following the initial publication by Peirce and colleagues [52], Tabibian and colleagues [77] published the second cell-based model of vascular calibre determination. In this paper, Tabibian and colleagues [77] presented a self-propelled particle model to understand endothelial cell behaviour during developmental remodelling in a quail embryo. The capillaries and the surrounding tissue were described using a population of discrete elliptical agents, and the initial configuration of the network within the local tissue was extracted from experimental images (Fig 8B). The migration of these elliptic agents was driven by (1) the local wall shear stress, (2) the attraction/repulsion body forces, and (3) the body forces perpendicular to the vessel walls. The relationship between the wall shear stress and the migration velocity was extracted from experimental data, which interestingly suggested a bell curve–like relationship. These data conflict with the assumption that the vessel radius is logarithmically dependent on the wall shear stress [15,61,62]. While the relationship between elliptical agents and endothelial cells was unclear (Fig 8B), the simulated networks were shown to have a Hausdorff distance of 46.4 to 52.2 *μm* when compared to experimental images. The authors used this model to show that the wall shear stress had a greater impact than body forces on remodelling and that the wall shear stress caused elongation of the elliptical agents.

More recently, Edgar and colleagues [78] published a cell-centre cell-based model to examine how endothelial behaviour determines branch selection and vessel stability at bifurcations. In particular, this model was developed to provide a theoretical understanding of the role of junction force transmission between endothelial cells during remodelling and to discern the importance of these forces as a mechanism to avoid functional shunting. In this model, endothelial cells were represented as elliptic agents and arranged across the lumen of a network consisting of a feeding vessel and a draining vessel, connected by a proximal branch and a distal branch (forming an “A”-shaped bifurcation). Each vessel was discretised into luminal segments, and the radius of these segments was calculated by the number of endothelial cells contained in the segment. Endothelial cells migrated against the flow, which was calculated using the Hagen–Poiseuille equation at each time step. Upon approaching a bifurcation, cells chose a vessel according to a weighted probabilistic function, which was dependent on both the number of cells and the wall shear stress in each daughter vessel. Using this model, the authors found that the flow-based and collective-based migratory cues were equally important. Were one factor expressively favoured, shunt formation was more likely and occurred far earlier.

Shortly after this initial publication by Edgar and colleagues [78], the authors extended the cell-centre model of endothelial cell behaviour to a larger honeycomb network and introduced a more explicit expression for intracellular adhesion forces [79]. In this model, endothelial cells were defined over the luminal surface of a 3D honeycomb network, and movement along the surface was restricted to discrete steps between discrete cylindrical luminal segments. Again, the cells migrated against the flow at a constant speed, but now the adhesive forces between cells was modelled using electrostatic-like attraction-repulsion forces. Using this model, the authors showed that the evolution of the developing network was highly sensitive to the cellular adhesive forces. Where the adhesion forces were high, cells tended to aggregate along a fraction of the flow pathways through the network, thereby depleting cells from other pathways and promoting the formation of functional shunts. Alternatively, when the adhesion forces were lower, a more homogeneous distribution of cells across the network was reported. A similar effect was seen by increasing the repulsion forces between cells, which was shown to increase migration speed, and promoted a uniform cell coverage. These results are contrary to those shown by Tabibian and colleagues [77], where it was reported that wall shear stress had the greatest impact on cellular behaviour. This discrepancy is likely a result of the different scales considered and the difference in agent size; Edgar and colleagues [78,79] considered each agent to represent a cell (Fig 8C), while the relationship between cells and agents in the Tabibian model was unclear, and agents were only a fraction of the size (7 *μm* radius) of an endothelial cell (Fig 8B).

Cell-based models of remodelling are in their infancy and have the capacity to significantly extend our theoretical understanding of vascular remodelling. The models discussed in this section are very recent developments in the field (for the most part) and have laid the foundations of a paradigm that is likely to become a prominent modelling approach in coming years. A key strength of cell-based models is their ability to explicitly incorporate heterogeneities within and between cellular populations [97]. This functionality must be leveraged to produce a clearer understanding of the many heterogeneous factors and processes driving remodelling. Several notable examples of these heterogeneities that should be considered include endothelial cell phenotype [1,98–100], shape [1,9,101–104] and polarity [8,13], variations in the local mechanical [105–107] and molecular [6,19,21,26,108] environments, the surrounding cellular populations [64,109], and the determination of vascular identity [1,10] (Fig 4: Row 3).

In each of the studies discussed in Sections 2 to 4, blood flow was explicitly modelled as a Newtonian fluid to determine the wall shear stress throughout the network. Most of these studies modelled blood flow using a simple network-based model, assuming the flow to be Hagen–Poiseuille flow [52,61,72,73,76,78,79]. In these models, the flow through each segment is solved simply through the following set of equations:

$$Q_{i}=\frac{\pi r_{i}^{4}\Delta P_{i}}{8L_{i}\eta_{i}},and\;\tau_{w,i}=\frac{D_{i}\Delta P_{i}}{4L_{i}},$$ (2)

where *Q*_*i*_, *r*_*i*_, *P*_*i*_, and *L*_*i*_ are the flow, radius, pressure, and length of vessel segment *i*. The wall shear stress in vessel segment *i* is given by *T*_*w*,*i*_. The apparent viscosity, *η*_*i*_, is a function of the local blood haematocrit and diameter. This is generally calculated using the expression given in Pries and colleagues [110] constructed from experimental data. Tabibian and colleagues [77] adopted a different approach, assuming the blood flow to be Stokes flow and opted to use a finite element method to solve the flow through the network, providing a greater degree of spatial resolution within each vessel. The blood flow through the vascular network plays a primary role in vascular regression [14,16,17,20,105,106,111]. As such, accurate models of flow through the network are necessary to provide a detailed understanding of flow driven regression and are discussed in detail in the following section.

## 5 Modelling paradigm 4: Flow-focused models of regression

Blood flow is an important driver of vascular remodelling and regression [14,16,17,20,105,106,111]. Much of what we know about flow-driven remodelling has been gained through computational models of blood flow through biologically realistic vascular networks [7,8,13,15,18,80,81]. This particular area of mathematical biology is an excellent example of where an integrated experimental/mathematical approach has driven the field forwards.

**Fig 9:**
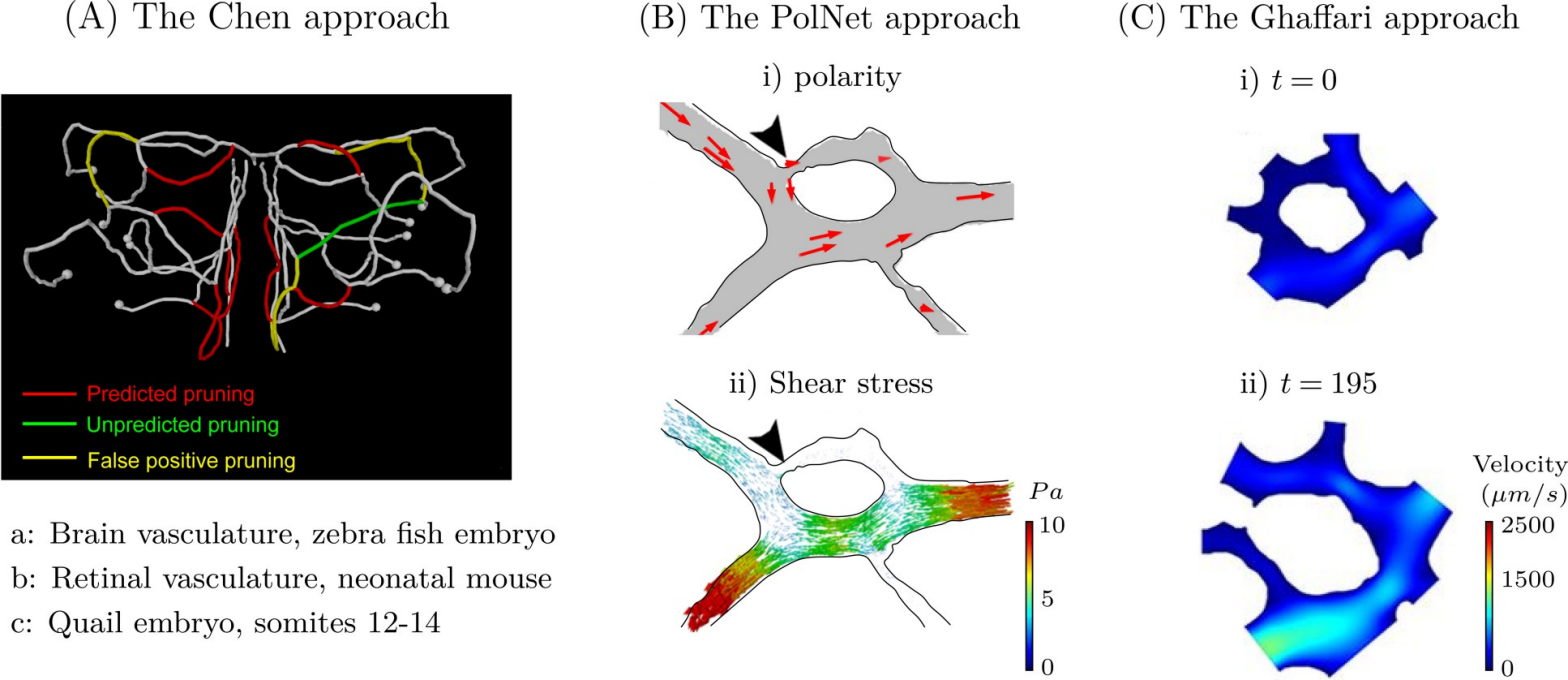
The Chen (A), PolNet (B), and Ghaffari (C) approaches are the 3 flow-focused models of vascular remodelling currently available in the literature. (**A**) Chen and colleagues [15] coupled long-time series confocal imaging of the developing zebrafish midbrain vasculature (in vivo) with a fluid-structure interaction model to predict which vessels would undergo pruning during vascular development. (**B**) The PolNet group [7,8,13,18] compared live in vivo images of the retinal vasculature in neonatal mice with a 3D lattice-Boltzmann reconstruction of the blood flow to show that endothelial cell polarity (bi) aligns against the direction of flow, with polarisation strength inversely proportional to the wall shear stress (Bii). (**C**) Ghaffari [80,81] coupled time-lapse microscopy images of flow dynamics in ex ovo quail embryos with a finite element model of the 2D Navier–Stokes equations to understand how the flow evolves during development. Subfigures A, B, and C are adapted with permission from Chen and colleagues [15], Franco and colleagues [13], and Ghaffari and colleagues [80], respectively.

Currently, there are 3 main approaches to modelling flow through the developing vasculature: the Chen approach [15], the PolNet approach [7,8,13,18], and the Ghaffari approach [80,81], shown in Fig 9.

In recent years, several groups have pioneered an approach to study the haemodynamic forces on the vessel wall by coupling experimental techniques with computational fluid dynamics (Figs 5 and 9). The first to do this was Chen and colleagues [15], who coupled long-time series confocal imaging of the developing zebrafish midbrain vasculature in vivo with a simple fluid-structure interaction model of vessel development (Fig 9A). Chen and colleagues [15] approximated the capillaries extracted from the confocal images as 2D line segments, simulated blood flow through the network using a simple network model, and assumed the vessel radius was linearly dependent on the local wall shear stress. The network geometry and flow profile were updated iteratively as selected vessels collapsed and regressed until a steady state was achieved. Upon comparison with experimental results, the model was able to predict vessel regression with 75% success. Moreover, a significant flow reduction was noted in the hour preceding the decrease in diameter in regressing vessels. As such, the authors suggested that changes in the blood flow triggers vessel regression. The authors went further to examine the effect of pulsatile flow to show that regression occurred more rapidly; however, there were no changes observed in which vessels regressed.

Shortly following the initial flow-focused model of regression published by Chen and colleagues [15], the PolNet group [7,8,13,18] published a series of articles implementing a high-resolution 3D lattice-Boltzmann reconstruction of the haemodynamic environment through realistic vascular networks using HemeLB [7,8,13,18] (Fig 9B). Three-dimensional reconstructions of the retinal vascular network where extracted from confocal images, taken from neonatal mice and the full 3D Navier–Stokes equations were then solved throughout the network using the lattice-Boltzmann method. Using this computational framework, Bernabeu and colleagues [7] showed that vascular regression occurred in regions of the network with notable wall shear stress gradients. The wall shear stress was notably lower in vessels undergoing, or about to undergo, regression, while higher wall shear stress values were observed in regions of the network at a more advanced state of remodelling. These simulation results are in good agreement with those presented by Chen and colleagues [15]. Going further, Bernabeu and colleagues [7] noted important differences in the flow patterns across the retinal network over consecutive days, highlighting that preferential paths begin to emerge over consecutive days in the un-remodelled parts of the network. At postnatal day 5, the wall shear stress distribution was more uniform across the network, while by postnatal day 6, significant variations in wall shear stress had emerged. Wall shear stress was notably higher through the arterioles, venules, and across the remodelled sections of the network, compared to the sprouting front, where the wall shear stress was still low and homogeneously distributed.

In a subsequent paper from this group, Franco and colleagues [13] extended upon the model presented in Bernabeu and colleagues [7], by coupling it with a live imaging in vivo study to show that endothelial cells polarize against the flow. Endothelial cell polarisation was shown to be stronger in regions with lower wall shear stress values, and the cells were shown to migrate in the direction of polarization (Fig 9B). Moreover, it was shown that the dynamic and polarized migration of endothelial cells lead to the regression of vessel segments experiencing low flow, while vessel segments experiencing high flow were stabilised.

More recently, the PolNet group have extended their lattice-Boltzmann model of blood flow to explicitly incorporate hyperelastic red blood cells using an immersed boundary method [66,82,83,90]. The incorporation of deformable red blood cells provides a more realistic model of the mechanical forces acting on the developing vessel walls and has advanced our understanding of vessel selection during flow driven regression. Using this model, Zhou and colleagues [66] demonstrated that the explicit incorporation of deformable red blood cells produced larger wall shear stress gradients between vessels about to regress and the surrounding vessels than had previously been seen over the same networks using a single-phase lattice-Boltzmann model of blood flow [7].

Moreover, this explicit consideration of red blood cells suggests that vessels devoid of red blood cells maybe more likely to regress [66]. As such, this model presents evidence that the redirection of red blood cells has an important role in flow-driven vascular regression. By coupling experimental imaging results with lattice-Boltzmann simulations of fluid flow, the PolNet group have provided a deeper understanding of the role of haemodynamics during vascular development, beyond that which is measurable through a purely experimental approach.

In 2015, Ghaffari and colleagues [80,81] adopted a different approach to model the flow dynamics through the evolving vasculature of the quail embryo. Ghaffari and colleagues [80,81] developed a technique to simultaneously image vascular development and flow dynamics using time-lapse microscopy in the capillary plexus of ex ovo quail embroys during vascular remodelling. Flow through the network was traced using fluorescent microspheres and the velocity of the microspheres was calculated using a technique referred to as microparticle image velocimetry (*μ*PIV). However, the authors found that *μ*PIV only provided accurate measurements of velocity within straight vessel segments [80,81]. As such, Ghaffari and colleagues [80,81] used a finite element method to solve the 2D Navier–Stokes equations over the discretised microscopy images and used the *μ*PIV calculations as Neumann boundary conditions (Fig 9C). Using this technique, the authors confirmed that vessel segments experiencing a low wall shear stress are more likely to regress [81]. These 2 papers by Ghaffari and colleagues [80,81] are the only ones to simulate flow through the domain over many sequential time-steps, enabling the authors to consider how the fluid flow evolves over time. Ghaffari and colleagues [80] went further to examine the relationship between wall shear stress and change in vessel diameter in vascular networks from 3 separate quail embryos, showing that the logarithm of the change in vessel diameter was correlated (*r* = 0.75) with wall shear stress. This result supports the hypothesised logarithmic relationship between diameter and wall shear stress first proposed by Pries and colleagues [61].

Current efforts in the PolNet group are focused on extending their existing models to 3D and further utilising their immersed boundary model of red blood cells (Fig 4: Row 4). A major challenge for flow-based models in the coming years will be integrating this modelling paradigm with the other paradigms, specifically with cell-based models of remodelling. Such an extension will help us understand not only how the endothelial cells respond to more realistic models of flow but also how the flow evolves as the network adapts and remodels in response to the flow-based stimuli.

## 6 Future development

**Fig 10:**
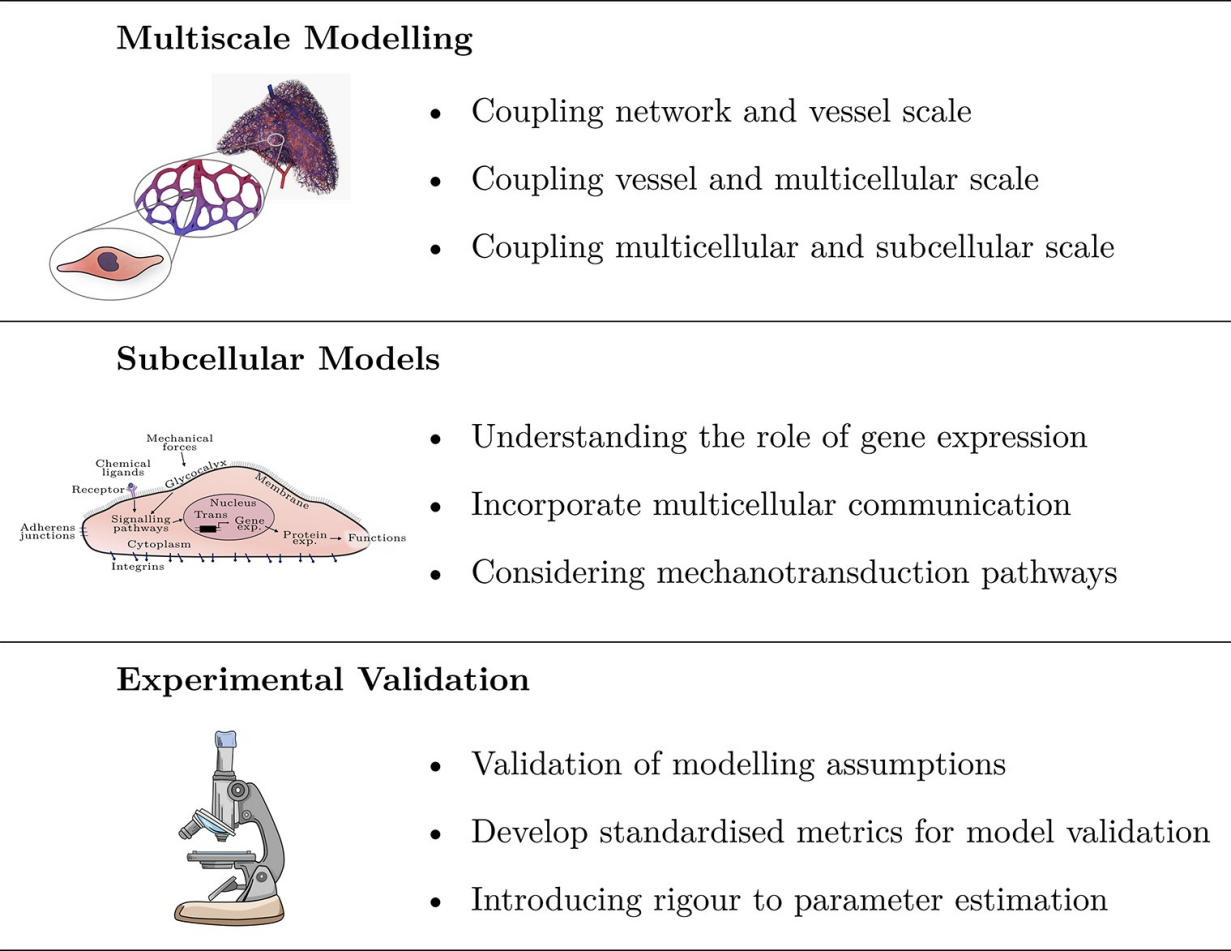
Key challenges for the development of mathematical models of vascular remodelling. The 3D printed vascular network shown on the top row has been reproduced with permission from [11].

Mathematical models of developmental vascular remodelling are only beginning to emerge. Over the past 40 years, most mathematical models examining vascular development have focused on angiogenesis. As such, there is still much work to do in the field of mathematical models for developmental vascular remodelling, and there are many biological questions yet to address. The existing models of vascular remodelling have provided a stable bedrock from which to expand into this yet uncharted territory. In this section, we will address several key challenges for the field, each of which are outlined in Fig 10.

### 6.1 Challenge 1: Development of multiscale and multiphysics models of developmental vascular remodelling

Vascular remodelling is inherently a multiscale phenomenon (Fig 2). The complexity of vascular development presents many challenges in the establishment of an integrated multiscale model of remodelling. This complexity emerges from (1) the large number of processes occurring on each scale, (2) the strong interdependence between each scale, and (3) the multiphysics nature of development [1,6,12]. In particular, the multiphysics nature of vascular development introduces modelling challenges requiring the coupling of separate fields of applied mathematics, such as continuum mechanics and systems biology. This challenge will require a considerable breadth of knowledge, large collaborations, and currently unattainable experimental data. Despite the apparent challenges, coupling scales will overcome many limiting assumptions plaguing today’s models.

1. **Coupling network and vessel scales:** The largest spatial jump between adjacent scales will be that between the vessel and network scales (~10 *μm* to ~10 *mm*) [6]. At current, there are no models considering remodelling at a network, or organ level, and this remains an ongoing challenge for the field. There is a need to consider the optimisation processes in which oxygen delivery throughout the tissue is maximised while energy expenditure from the vascular system is minimised at a network level. While this model is necessary for an organ level understanding of remodelling, consideration of the spatially organised processes occurring at the vessel scale are necessary. As such, a judiciously coupled multiscale approach will be required to minimise computational complexity.
2. **Coupling vessel and multicellular scales:** Multiscale coupling between the vessel scale and the multicellular scale will see significant advances in currently available modelling approaches. Models considering remodelling at the vessel scale (hybrid models and logarithmic models; Sections 2 and 3) assume each vessel responses in a uniform manner and disregard the important role of the both the individualised and collective cellular behaviour [61,62,65,67–76]. Changes in vessel diameter and structure are born from cellular rearrangement, with cells moving between vessels [12]. Moreover, the cellular state dictates the vessel identity determination and vascular stabilisation, which both have important implications for vascular calibre control [1]. Current vessel-level models ignore this direct relationship between neighbouring vessels and the complexities occurring at the multicellular scale, thereby introducing biases into the model and possibly concealing important biological processes. As such, linking the multicellular and vessel scale is a necessary step for the field. However, this will come at the cost of model complexity and introduce increased computational loads.
3. **Coupling multicellular and subcellular scales:** Endothelial cells behaviour is governed by processes occurring at the subcellular level. Existing models of remodelling at the multicellular scale (Section 4) assume that the endothelial population responds in a spatially and temporally uniform manner [52,78,79]; however, experimental studies show endothelial behaviour can vary significantly both within and between populations [45,46,112–115]. Integrating the subcellular processes occurring within each cell with a larger cell-based model will be critical in providing a rigorous description of remodelling. This coupling will be challenging due to both the complexity of the subcellular processes and the absence of subcellular models of remodelling. The need for subcellular models of remodelling is discussed in further detail in the following paragraphs.

### 6.2 Challenge 2: Development of subcellular models of endothelial cells during developmental vascular remodelling

The absence of models at the subcellular scale is a nontrivial issue; processes at this scale drive development at the larger scales. While experimental studies are beginning to decipher these subcellular processes, there is still much unknown about how these processes generate network-level organisation and optimisation. Subcellular processes govern the behaviour of endothelial cells, including how they respond to mechanical and chemical stimuli and multicellular interactions [1,12]. Although there are thousands of experimental studies exploring many of these individual subcellular process (e.g., [112,116–123]), it is still difficult to resolve how these pathways interact and how they work in concert to produce vascular remodelling using the currently available experimental techniques [1]. As such, there is still much unknown about how subcellular decisions generate network-level hierarchical organisation and optimisation. Thusly, there is a clear need for the development of mathematical models of the subcellular processes driving remodelling. In particular, there is a specific need to develop subcellular models to understand:

1. **Understanding the role of gene expression:** Gene expression dictates how the cell responds to external cues and stimuli [124]. Moreover, gene expression drives the determination of the endothelial cell phenotype and organotypic assignment [44]. The endothelial cell phenotype plays a large role in dictating the response the cells have to external cues, as such deliberation and understanding of cell phenotype and phenotype determination is important to understand the cellular responses to the stimuli driving remodelling [1]. In doing so, this will move away from the assumption that endothelial cells are a homogeneous population.
2. **Incorporate multicellular communication:** The endothelial cells forming the capillary wall are connected by several proteins, such as such as adherens junctions [125]. These connecting proteins facilitate commutation between neighbouring cells and allow the transmission of mechanical and chemical signals, altering the cellular behaviour and synchronising endothelial cell population responses and behaviours [126]. These signal transduction pathways between cells are important to understand how collections of cells make decisions due to the subcellular structures and processes [127].
3. **Considering mechanotransduction pathways:** Cellular mechanotransduction is a complicated processes with many pathways through which mechanical information is transferred into the cell [128,129]. External forces trigger conformational changes within the cell, influence cellular behaviour, and can alter gene expression [129]. These processes are further complicated by the modulating role of both cellular signalling factors and communication pathways between neighbouring cells [130]. The establishment of subcellular mathematical models of remodelling will extend our understanding of how cells respond to these forces at a subcellular level. Moreover, the consideration of mechanotransduction pathways, along with the modulating role of cellular signalling, may provide a more detailed understanding of flow-driven regression.

### 6.3 Challenge 3: Experimental validation of existing and future models

The models currently available in the literature provide a valuable insight into remodelling and have established a considerable foundation to build upon. However, a notable issue with many existing models is the lack of experimental validation of both the model assumptions and the model results. This lack of experimental validation has several root causes, namely, (1) the high degree of biological variation; (2) the absence of standardised metrics for model validation; and (3) noisy or incomplete experimental data.

1. **Validation of modelling assumptions:** Subcellular processes induce a high degree of variation between vascular networks (both within an individual and between individuals) [118,131]. As such, comparing model results with a “standard” or typical experimental network can be challenging. This challenge is amplified by the simplifying modelling assumptions often made, which ignore, simplify, or homogenise these subcellular processes. The modelling assumptions themselves are often unverified or unvalidated, risking the propagation of uncharacterised artefacts throughout the results. As such, validating model assumptions is of prime importance.
2. **Develop standardised metrics for model validation:** Moving forward, it is necessary to establish standardised metrics with which to compare model results with experiential data. Such metrics could consider the complexity of the network, vessel characteristics (such as length and bifurcation angles), and characteristics of the endothelial cell population (such as cell number, shape, polarity, and phenotype).
3. **Introducing rigour to parameter estimation:** In addition to model and assumption validation, model parameter estimation is an important factor often overlooked. While it is difficult to derive parameter estimates or distributions from experimental data, it is still important to understand how perturbations in parameter selection impacts the model. This point is simply addressed by introducing a sensitivity analysis; however, this practice is yet to be regularly adopted by the mathematical vascular development community. The introduction of standardised metrics will provide a pathway to consider parameter optimisation, model selection, test modelling assumptions, and validation of the model.

The field of mathematical modelling of developmental vascular remodelling is in its infancy. The existing models have made important contributions to our understanding of vascular remodelling; however, there is much work yet to be done. Several key future directions are identified in this article: (1) the development of a multiscale and multiphysics approach; (2) the development of subcellular models; and (3) experimental validation of existing and future models.
